# Urinary Bisphenol A and Hypertension in a Multiethnic Sample of US Adults

**DOI:** 10.1155/2012/481641

**Published:** 2012-01-27

**Authors:** Anoop Shankar, Srinivas Teppala

**Affiliations:** Department of Community Medicine, West Virginia University School of Medicine, P.O. Box 9190, Morgantown, WV 26506-9190, USA

## Abstract

*Background*. Bisphenol A (BPA) is a common chemical used in the manufacture of polycarbonate plastics and epoxy resins, with >93% of US adults having detectable BPA levels in urine. Recent animal studies have suggested that BPA exposure may have a role in several mechanisms involved in the development of hypertension, including weight gain, insulin resistance, thyroid dysfunction, endothelial dysfunction, and oxidative stress. However, no previous human study has examined the association between markers of BPA exposure and hypertension. *Methods*. We examined urinary BPA levels in 1380 subjects from the National Health and Nutritional Examination Survey 2003-2004. Main outcome-of-interest was hypertension, defined as blood pressure-reducing medication use and/or blood pressures >140/90 mm of Hg (*n* = 580). *Results*. We observed a positive association between increasing levels of urinary BPA and hypertension independent of confounding factors such as age, gender, race/ethnicity, smoking, body mass index (BMI), diabetes mellitus and total serum cholesterol levels. Compared to tertile 1 (referent), the multivariate-adjusted odds ratio (95% confidence interval) of hypertension associated with tertile 3 was 1.50 (1.12−2.00); *P*-trend = 0.007. The association was consistently present in subgroup analyses by race/ethnicity, smoking status, BMI, and diabetes mellitus. *Conclusions*. Urinary BPA levels are associated with hypertension, independent of traditional risk factors.

## 1. Introduction

Bisphenol A (BPA) is a chemical produced in very high volume worldwide [1]. It is used extensively in the manufacture of epoxy resins, polycarbonate plastics, and food and beverage containers and also in cigarette filters and carbonless copy paper used in credit card receipts and thermal imaging papers in modern cash registers [1]. Consequently BPA is one of the commonly exposed environmental chemicals in humans [2]. Detectable levels of BPA in urine have been shown to be present in nearly all sampled adults in recent national surveys conducted in the USA [2, 3].

BPA is considered to be an endocrine-disrupting chemical and has been shown to have thyroid hormone disrupting effects [1, 4]. Recent evidence, especially from experimental animal studies, suggests that BPA exposure may be related to insulin resistance and may have a role in weight gain and obesity [5, 6]. Furthermore, recent epidemiological studies have shown that higher urinary BPA levels are related to diabetes mellitus [7] as well as to self-reported cardiovascular disease [8, 9]. Recent studies have also shown that BPA exposure is related to hyperlipidemia [10], as well as to increased levels of serum markers of oxidative stress [11], inflammation [11].

Hypertension is a major public health problem, and it is also known to be a strong, independent risk factor for CVD [12]. Several of the biological mechanisms that are known to be associated with increased risk of hypertension, including thyroid dysfunction [13], weight gain [14], insulin resistance [15], hyperlipidemia [16], oxidative stress [17], and higher systemic inflammation [18], are also related to increased risk of developing hypertension. However, no previous study to our knowledge has examined the association between BPA levels and hypertension in humans. Therefore, we examined the independent association between urinary BPA levels and hypertension in the 2003-2004 National Health and Nutritional Examination Survey (NHANES), a contemporary, multiethnic sample of US adults.

## 2. Methods

The current study is based on data from the NHANES 2003-2004. Detailed description of NHANES study design and methods are available elsewhere [19]. In brief, the NHANES survey included a stratified multistage probability sample representative of the civilian noninstitutionalized US population. Selection was based on counties, blocks, households, and individuals within households and included the oversampling of non-Hispanic blacks and Mexican Americans in order to provide stable estimates of these groups. Subjects were required to sign a consent form before their participation, and approval was obtained from the Human Subjects Committee in the US Department of Health and Human Service.

The current study sample consisted of participants aged greater than 20 years among whom urinary BPA was available (*N* = 1, 488). We further excluded participants with missing data (*n* = 108) on covariates included in the multivariable model, including level of education, smoking status, serum or fasting glucose levels, body mass index (BMI), and cholesterol levels. This resulted in 1380 participants (49.6% women), 580 of whom had hypertension.

### 2.1. Exposure Measurements

Age, gender, race/ethnicity, smoking status, alcohol intake (g/day), level of education, history of diabetes and oral hypoglycemic intake or insulin administration were assessed using a questionnaire [19]. Individuals who had not smoked >100 cigarettes in their lifetimes were considered never smokers; those who had smoked >100 cigarettes in their lifetimes were considered former smokers if they answered negatively to the question “Do you smoke now?” and current smokers if they answered affirmatively. Body mass index (BMI) was calculated as weight in kilograms divided by height in meters squared.

Rigorous procedures with quality control checks were used in blood collection, and details about these procedures are provided in the NHANES Laboratory/Medical Technologists Procedures Manual [19]. Serum glucose was measured using the modified hexokinase method at the University of Missouri Diabetes Diagnostic Laboratory. Diabetes mellitus was defined based on the recent guidelines of the American Diabetes Association [20] as a serum glucose >126 mg/dL after fasting for a minimum of 8 hours, a serum glucose >200 mg/dL for those who fasted <8 hours before their NHANES visit, a glycosylated hemoglobin value >6.5%, or a self-reported current use of oral hypoglycemic medication or insulin.

Previous measures of BPA in biological matrixes involved techniques such as gas chromatography (GC) or high-performance liquid chromatography [21]. To achieve enhanced sensitivity and selectivity over previous methods, in the current NHANES, measures of environmental phenols were derivatized to alkyl or acyl derivatives before GC/mass spectrometry (GC/MS) analysis [22]. Using solid phase extraction coupled to high-performance liquid chromatography and tandem mass spectrometry, detection levels of 0.1–2 nanograms per milliliter (ng/mL) in 100 *μ*L of urine were achieved, sufficient for measuring urinary BPA levels in nonoccupationally exposed participants [22].

### 2.2. Main Outcome of Interest: Hypertension

Seated systolic and diastolic blood pressures were measured using a mercury sphygmomanometer according to the American Heart Association and the Seventh Joint National Committee (JNC7) recommendations [12]. Up to 3 measurements were averaged for systolic and diastolic pressures. Patients were considered hypertensive if they reported current blood-pressure-reducing medication use and/or had systolic blood pressures >140 mm of Hg and/or diastolic blood pressures >90 mm of Hg [19].

### 2.3. Statistical Analysis

Urinary BPA was categorized into tertiles (<1.5 ng/mL, 1.5–4.0 ng/mL, >4.0 ng/mL). We hypothesized that high BPA levels are associated with hypertension. The odds ratio ((OR) (95% confidence interval (CI)) of hypertension associated with higher BPA levels was calculated using multivariable logistic regression models by taking the lowest tertile as the referent. We used two models: the age, sex-adjusted model and the multivariable model, additionally adjusting for race/ethnicity (non-Hispanic whites, non-Hispanic blacks, Mexican Americans, others), education categories (below high school, high school, above high school), smoking (never smoker, former smoker, current smoker), alcohol intake (nondrinker, moderate drinker, heavy drinker), BMI (normal, overweight, obese), hypertension (present, absent), diabetes (present, absent), and total serum cholesterol (mg/dL). Trends in the OR of hypertension across increasing urinary BPA categories were determined by modeling BPA as an ordinal variable. Sample weights that account for the unequal probabilities of selection, oversampling, and nonresponse were applied for all analyses using SAS (version 9.2; SAS Institute, Cary, NC) and SUDAAN software; SEs were estimated using the Taylor series linearization method.

## 3. Results

Table 1 shows the baseline characteristics of the population. Overall, this was a middle-aged multiethnic sample with approximately 32% subjects obese, 10% subjects having diabetes mellitus, and 36.3% % having hypertension.

Table 2 shows the association between increasing levels of BPA and hypertension. Overall, we observed a positive association between increasing BPA levels and hypertension in both the age, sex-adjusted model and the multivariable-adjusted model. Models evaluating trend in this association were also statistically significant.

Table 3 shows the association between increasing BPA levels and hypertension within subgroups of race/ethnicity, BMI, and diabetes status. We found that the positive magnitude of association between increasing BPA levels and hypertension was consistently present within all these various stratified subgroups with the multivariable OR ranging from 1.4 to 1.9. Also, there was no statistically significant interaction between BPA levels and these variables (*p*-interaction >0.10 in all stratified analyses).

 We also performed several supplementary analyses. First, we examined the association between urinary BPA and hypertension using urinary BPA as a continuous variable (with natural logarithmic transformation). Consistent with the categorical BPA analysis, there was a significant positive association between urinary BPA as a continuous variable and hypertension with an odds ratio (95% CI) of 1.18 (1.07–1.31); *P* value = 0.001 in the age-sex-adjusted model and 1.11 (1.01–1.22); *P*-value = 0.04 in the multivariable model for every 1 unit increase in the log-transformed urinary BPA level. Second, to examine the nonlinearity of the association between urinary BPA and hypertension, we added a quadratic term for BPA in the multivariable logistic regression models. We found that the quadratic term was not statistically significant (*P*-value = 0.459 in the age-, sex-adjusted model and 0.784 in the multivariable-adjusted model).

## 4. Discussion

In a large multiethnic, representative sample of US adults, we found that increasing urinary BPA levels were associated with hypertension. The observed association was found to be independent of confounding factors such as smoking, BMI, alcohol intake, diabetes mellitus, and serum cholesterol level. Our study adds to the emerging evidence of the role of environmental exposure to BPA on health outcomes in humans [8, 9] by being the *first study *reporting an association between BPA exposure and hypertension, which is a major public health condition and a strong risk factor for CVD [12].

 BPA is an environmental chemical used as a constituent monomer in polycarbonate plastics, which are used extensively in drinks containers and food packaging, and in the production of oxidants used in the lining of canned goods [1]. Exposure to BPA is believed to be mainly through dietary intake with additional exposure through water, dental sealants, inhalation of household dusts, and exposure through skin [1]. Recent studies have documented that over 93 to 95% of the general population has measurable concentrations of BPA metabolites in urine [2, 3].

 Several lines of recent evidence suggest that an association between urinary BPA levels and hypertension may be biologically plausible. Animal studies have suggested that BPA exposure may have a role in the development of hypertension thorough several mechanisms, including BPA's role in weight gain and obesity development potentially through its actions on preadipocytes [23, 24], role as an estrogen [5], potential interactions with estrogen-related receptor gamma [25], actions as a thyroid hormone antagonist [4], role as a peroxisome proliferator-activated receptor gamma antagonist [26], and its role in influencing pancreatic endocrine function [27]. Alonso-Magdalena et al. [28] in a recent experiment showed that mice exposed to long-term exposure to BPA developed hyperinsulinemia, insulin resistance, and glucose intolerance. Furthermore, BPA has been shown to induce endothelial cell injury mediated through oxidative stress [29–31] and elevations in lipids [32] in animal models. In epidemiological studies in humans, BPA levels were found to be associated with abnormal liver function enzymes, higher levels of fasting glucose, insulin, and HOMA-IR insulin resistance [8, 9], all are factors known to be associated with hypertension development [15, 33].

 However, there are no previous studies in humans for comparison. It is in this context that our results regarding objectively measured systolic and diastolic blood pressures to define hypertension in a large sample of US adults are important. In the current study, we have demonstrated a consistent, positive association between higher BPA levels and hypertension that was independent of major confounders and also consistent in stratified analyses by race/ethnicity, BMI, and diabetes mellitus.

 The main strengths of our study include its nationally representative sample, use of rigorous study methods to collect the data, and the availability of extensive data on confounders [19, 22]. The main study limitation is that the current study is cross-sectional in nature, making it impossible to draw cause-effects in the observed associations. Future prospective studies are required to confirm or disprove our findings.

 In summary, we found that in a nationally representative sample of US adults, higher BPA levels were positively associated with hypertension independent of confounding factors such as age, gender, smoking, BMI, alcohol intake, diabetes mellitus, and cholesterol levels. If confirmed in future prospective studies, reducing environmental exposure to BPA may have a role in the prevention of hypertension.

## Figures and Tables

**Table 1:** Baseline characteristics of the study population*.

Characteristics	Whole cohort (*n* = 1380)
Age (years)	46.2 + 0.5
Female (%)	700 (49.6)
Race/Ethnicity (%)	
Non-Hispanic Whites	748 (72.3)
Non-Hispanic Blacks	244 (9.8)
Mexican Americans and others	388 (17.8)
Education categories (%)	
Below high school	395 (17.9)
High school	331 (25.3)
Above high school	654 (56.8)
Smoking (%)	
Never smoker	691 (50.6)
Former smoker	373 (25.5)
Current smoker	316 (23.9)
Alcohol intake (%)	
Non-drinker	544 (34.1)
Moderate drinker	528 (41.7)
Heavy drinker	308 (24.0)
Body mass index (BMI) (%)	
Normal weight (<25 kg/m^2^)	448 (35.0)
Overweight (25–30 kg/m^2^)	475 (32.9)
Obese (BMI ≥ 30 kg/m^2^)	457 (32.1)
Diabetes (%)	175 (10.0)
Total cholesterol (mg/dL)	203.81 ± 1.17
Hypertension (%)	580 (36.3)

*Data presented are number (percentages) or mean values ± standard error (SE), as appropriate for the variable.

**Table 2:** Association between urinary bisphenol A and hypertension.

Bisphenol A tertiles (ng/mL)	Sample size (cases)	Age-, sex-adjusted OR (95% CI)*	Multivariable-adjusted OR (95% CI)^∗†^
Tertile 1 (<1.5)	447 (180)	1 (referent)	1 (referent)
Tertile 2 (1.5–4.0)	473 (198)	1.27 (0.81–1.98)	1.11 (0.71–1.74)
Tertile 3 (>4.0)	460 (202)	1.74 (1.27–2.38)	1.50 (1.12–2.00)
*P* trend		0.0006	0.007

*OR (95% CI): Odds Ratio (95% Confidence Interval)

^†^Adjusted for age (years), gender (male, female), race-ethnicity (non-Hispanic whites, non-Hispanic blacks, Mexican Americans, others), education categories (below high school, high school, above high school), smoking (never, former, current), alcohol intake (nondrinker, moderate drinker, heavy drinker), body mass index (normal, overweight, obese), diabetes (present, absent), and total cholesterol (mg/dL).

**Table 3:** Association between urinary bisphenol A (BPA) and hypertension, by stratification variables.

Stratification category	Sample size (cases)	Multivariable OR (95% CI)^∗†^ comparing BPA tertile 3 versus Tertile 1	*p*-interaction
Race/ethnicity			
Non-Hispanic whites	748 (316)	1.42 (0.98–2.06)	
Non-Hispanic blacks	244 (121)	1.91 (0.44–8.33)	0.507
Mexican Americans and others	388 (143)	1.50 (0.60–3.72)	
Smoking status			
Non-smokers	691 (265)	1.62 (1.01–2.59)	0.932
Smokers	689 (315)	1.39 (0.74–2.62)	
BMI categories			
Nonobese	923 (334)	1.37 (0.91–2.06)	0.729
Obese	457 (246)	1.84 (0.85–3.99)	
Diabetes mellitus/Prediabetes			
Absent	896 (282)	1.30 (0.87–1.95)	0.494
Present	484 (298)	1.91 (1.08–3.37)	

*OR (95% CI): Odds Ratio (95% Confidence Interval)

^†^Adjusted for age (years), gender (male, female), race-ethnicity (non-Hispanic whites, non-Hispanic blacks, Mexican Americans, others), education categories (below high school, high school, above high school), smoking (never, former, current), alcohol intake (non-drinker, moderate drinker, heavy drinker), body mass index (normal, overweight, obese), diabetes (present, absent) and total cholesterol (mg/dL).
